# Workplace-based primary prevention intervention reduces incidence of hypertension: a post hoc analysis of cluster randomized controlled study

**DOI:** 10.1186/s12916-023-02915-6

**Published:** 2023-06-14

**Authors:** Zhen Hu, Xin Wang, Conglin Hong, Congyi Zheng, Linfeng Zhang, Zuo Chen, Haoqi Zhou, Yixin Tian, Xue Cao, Jiayin Cai, Runqing Gu, Ye Tian, Lan Shao, Zengwu Wang

**Affiliations:** 1 Division of Prevention and Community Health, National Center for Cardiovascular Disease, State Key Laboratory of Cardiovascular Disease, Peking Union Medical College & Chinese Academy of Medical Sciences, National Clinical Research Center of Cardiovascular Disease, Fuwai Hospital, No. 15 (Lin), Fengcunxili, Mentougou District, Beijing, 102308 China; 2 Department of Epidemiology, School of Public Health, Suzhou Medical College of Soochow University, Suzhou, 215006 China; 3 Department of Biostatistics, Peking University, Beijing, China; 4 School of Population Medicine and Public Health, Chinese Academy of Medical Sciences & Peking Union Medical College, Beijing, 100730 China

**Keywords:** Workplace-based, Multicomponent, Prevention interventions, Incidence of hypertension, Randomized controlled, Post hoc analysis

## Abstract

**Background:**

A workplace-based primary prevention intervention be an effective approach to reducing the incidence of hypertension (HTN). However, few studies to date have addressed the effect among the Chinese working population. We assessed the effect of a workplace-based multicomponent prevention interventions program for cardiovascular disease on reducing the occurrence of HTN through encouraging employees to adopt a healthy lifestyle.

**Methods:**

In this post hoc analysis of cluster randomized controlled study, 60 workplaces across 20 urban regions in China were randomized to either the intervention group (*n* = 40) or control group (*n* = 20). All employees in each workplace were asked to complete a baseline survey after randomization for obtaining sociodemographic information, health status, lifestyle, etc. Employees in the intervention group were given a 2-year workplace-based primary prevention intervention program for improving their cardiovascular health, including (1) cardiovascular health education, (2) a reasonable diet, (3) tobacco cessation, (4) physical environment promotion, (5) physical activity, (6) stress management, and (7) health screening. The primary outcome was the incidence of HTN, and the secondary outcomes were improvements of blood pressure (BP) levels and lifestyle factors from baseline to 24 months. A mix effect model was used to assess the intervention effect at the end of the intervention in the two groups.

**Results:**

Overall, 24,396 participants (18,170 in the intervention group and 6,226 in the control group) were included (mean [standard deviation] age, 39.3 [9.1] years; 14,727 men [60.4%]). After 24 months of the intervention, the incidence of HTN was 8.0% in the intervention groups and 9.6% in the control groups [relative risk (RR) = 0.66, 95% CI, 0.58 ~ 0.76, *P* < 0.001]. The intervention effect was significant on systolic BP (SBP) level (*β* =  − 0.7 mm Hg, 95% CI, − 1.06 ~  − 0.35; *P* < 0.001) and on diastolic BP (DBP) level (*β* =  − 1.0 mm Hg, 95% CI, − 1.31 ~  − 0.76; *P* < 0.001). Moreover, greater improvements were reported in the rates of regular exercise [odd ratio (OR) = 1.39, 95% CI, 1.28 ~ 1.50; *P* < 0.001], excessive intake of fatty food (OR = 0.54, 95% CI, 0.50 ~ 0.59; *P* < 0.001), and restrictive use of salt (OR = 1.22, 95% CI, 1.09 ~ 1.36; *P* = 0.001) in intervention groups. People with a deteriorating lifestyle had higher rates of developing HTN than those with the same or improved lifestyle. Subgroup analysis showed that the intervention effect of BP on employees with educational attainment of high school above (SBP: *β* =  − 1.38/ − 0.76 mm Hg, *P* < 0.05; DBP: *β* =  − 2.26/ − 0.75 mm Hg, *P* < 0.001), manual labor workers and administrative worker (SBP: *β* =  − 1.04/ − 1.66 mm Hg, *P* < 0.05; DBP: *β* =  − 1.85/ − 0.40 mm Hg, *P* < 0.05), and employees from a workplace with an affiliated hospital (SBP: *β* =  − 2.63 mm Hg, *P* < 0.001; DBP: *β* =  − 1.93 mm Hg, *P* < 0.001) were significantly in the intervention group.

**Conclusions:**

This post hoc analysis found that workplace-based primary prevention interventions program for cardiovascular disease were effective in promoting healthy lifestyle and reducing the incidence of HTN among employees.

**Trial registration:**

Chinese Clinical Trial Registry No. ChiCTR-ECS-14004641.

**Supplementary Information:**

The online version contains supplementary material available at 10.1186/s12916-023-02915-6

## Background

Hypertension (HTN) is the leading global preventable risk factor for cardiovascular disease (CVD) and premature death [1, 2]. It is estimated that approximately 1.56 billion adults worldwide will be diagnosed with HTN by 2025 [3]. The prevalence of HTN is high and increasing in China, from 13.6% in 1991 to 23.2% in 2012 [4, 5]. Practice has proved that primary prevention measures can effectively delay or avoid the occurrence of CVD risk factors such as HTN, thus reducing the incidence and mortality of CVD [6]. Studies showed that 40 to 70% of the decrease in cardiovascular mortality in the USA from 1980 to 2000 can be attributed to risk factor control [7]. Therefore, timely primary prevention intervention may be an economical and effective measure to reduce the occurrence and development of chronic non-communicable diseases such as HTN and CVD in China.

Employees account for more than 70% of the adult population [8], and their health status is of great importance to the stability and development of society. However, studies showed that the cardiovascular health status of occupational population worldwide is not ideal [9–13]. It is estimated that cardiovascular morbidity and death cost the workplace up to $120 billion annually in the USA [14]. Previous studies by our team showed that the prevalence of HTN was high among Chinese working population, but rates of awareness, treatment, and control were low [11]. Whether workplace-based primary prevention intervention of CVD can help workers develop a healthy lifestyle, improve their health level, and reduce the incidence of CVD among the Chinese working population, few studies to date have been designed [15].

From January 2013 to December 2014, we conducted a program in order to improve overall employees’ cardiovascular health and blood pressure (BP) control among employees with HTN using a multicomponent intervention strategy that combined workplace wellness program and guidelines-oriented HTN management intervention. In previous study, the effectiveness of BP control among employees with HTN was proofed [16]. In the present study, among the overall employees without HTN who received workplace-based primary prevention intervention only, the incidence of HTN and the adoption of healthy lifestyle were assessed by a post hoc analysis.

## Methods

### Study design

Our study was a post hoc analysis of cluster randomized controlled study, full details of study design and protocol have been published previously [16]. Briefly, twenty urban medical institutions [Chinese center for disease control and prevention (CDC) or class III hospitals] were selected as subcenters, each consisting of 2 to 4 workplaces, which were comparable in sector, ownership, size, economic level, and medical condition. These workplaces covered private enterprises in manufacturing industry, state-owned enterprises (mining, manufacturing, power supply, transport and post, and others), universities and research institutes, and were not medical institutions. Considering the intervention benefits and ethical issues, we took the workplace as the randomized unit and conducted a 1:2 cluster randomized controlled method in each subcenter, two workplaces randomly were selected as the intervention group and the other as the control group. The group randomization was undertaken by our statistician (Z.C.) in the coordinating center, who was not involved in the trial and was blind to the workplaces. Finally, 40 workplaces were randomly assigned to the intervention group and 20 to the control group. There were about 500 employees in each workplace and at least 50 eligible HTN patients. After randomization, the baseline survey was completed. Intervention was carried out separately for overall employees and HTN patients: primary prevention of CVD for overall employees. On the basis of primary prevention, HTN management for patients with HTN. The intervention lasted for 2 years, and participants completed standardized questionnaires at the beginning and end of the intervention under the guidance of the researchers.

### Participants

In our study, all employees in the workplace were potential participants. The inclusion criteria were as follows: (1) fixed employees of the workplace; (2) signed voluntarily a consent form to participate in the program within 2 years; (3) avoided to interrupt the participation due to long-term leave, going abroad, retirement or resignation within 2 years. And exclusion criteria were as follows: (1) acute myocardial infarction (< 3 months) and stroke (< 3 months) patients in the acute stage; (2) pregnant women, nursing mothers; (3) not cooperate (intelligence, hearing, physical activity barriers) significantly; (4) with severe disease, life expectancy less than 2 years; (5) medical personnel.

According to previous published study [16], the prevalence of HTN in the workplace was 25.2% [13]. Considering the actual size of the workplace and the dropout of the participants, a total of 45,000 source populations were planned to be enrolled at the time of study design to ensure a required sample size of hypertensive patients could be screened.

**Fig. 1:**
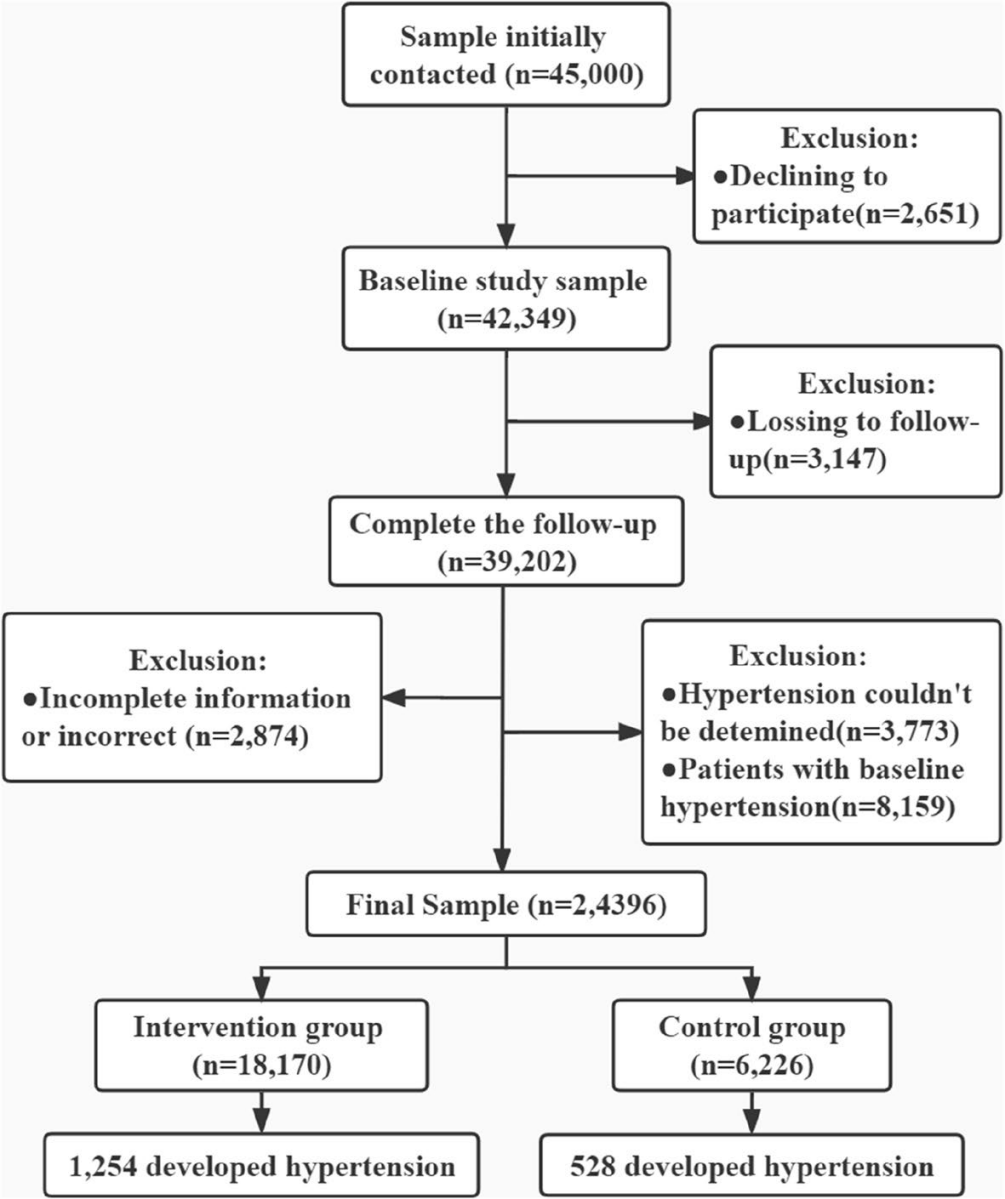
Flow chart of the participants selection process

Out of 42,349 employees who completed the baseline survey in 2012 with an overall follow-up rate of 92.57%, 24,396 employees were included in the final analysis after excluding 3147 employees who lost to follow-up (participants who did not participate in the last data collection), 2874 with incomplete information (participants whose first data cannot be matched with the last data), 3773 individuals whose HTN was not defined by existing data (variables necessary to define HTN were missing), and 8159 who had HTN at baseline (Fig. 1).

### Intervention

The intervention lasted 2 years with involving all participants. Based on the recommendations of the American Heart Association and the China Guidelines for Prevention of CVD [14], we developed a workplace-based primary prevention interventions program aimed at improving employees’ cardiovascular health, including (1) cardiovascular health education, (2) a reasonable diet, (3) tobacco cessation, (4) physical environment promotion, (5) physical activity, (6) stress management, and (7) health screening.

The health education included regularly organizing experts to give health lectures (at least half a year), putting up posters (quarterly), sending text messages, and distributing health education materials to educate employees on the risks of prevention of CVD. A reasonable diet included providing nutrition education and/or healthy eating information (e.g., control the total calories, limit the intake of oil, pay attention to the calorie distribution of three meals, nutrition balance) to employees and opening low-salt dishes windows (open at least one); affordable and healthy foods (e.g., low-salt, low-fat) were easily available, and participants were encouraged to make healthy food choices when eating lunch in the staff canteen, advising families to use as little cooking salt as possible (e.g., use a quantifiable salt spoon, try not to drink soup, reduce the amount of sodium-salt condiments such as glutamate, soy sauce, and so on). In addition, employees were encouraged not to drink alcohol as far as possible. If they do, a small amount of alcohol should be taken: the daily alcohol intake for women is less than 15 g/day, and for men, it is less than 25 g/day, no more than twice a week (15 g alcohol = 300 ml beer/150 ml wine/50 ml low-alcohol liquor). With regard to the smoking cessation section, we included disseminating the concept of smoking cessation, teaching methods and techniques for smoking cessation, distributing information pamphlets on smoking cessation, and establishing tobacco control regulations to increase smoking cessation rates among employees and to prohibit smoking in the workplaces. Improving the physical environment included modifying workstations and office layouts to reduce sedentary behavior and increase physical activity. The physical activity section encouraged participants to increase physical activity and advocated to actively fitness activities, for example, added sports facilities appropriately, encouraged to take elevators less and walk stairs more, organized and advocated various forms of physical activities such as workplace exercises, sports meetings, and regular group sports events (at least 2 items are completed). Accessible indoor or outdoor sports facilities, including indoor walking paths, were provided to allow employees in regular physical activities, encouraging employees to achieve their desired weight goals in a healthy way [body mass index (BMI) < 24 kg/m^2^; waist size: male < 90 cm, women < 85 cm]. For the stress management section, relaxation techniques were provided by the employees specialized in meditation, tai chi, or deep breathing for coping with stress monthly. The health screening section included annual health checkups and feedback to identify key risk factors. Employees in the control group were visited in the community health centers (CHCs) at baseline and the end of the program, receiving only routine care to prevention or treatment of diseases and receiving no any intervention from the program. The implementation for primary prevention had been published previously [16].

### Definitions and measurements

Baseline and 2-year follow-up data of intervention and control groups including information of sociodemographic characteristics, lifestyle behaviors, history of disease, and other factors were collected by trained health-care professionals at face-to-face visits using a standardized questionnaire developed by the coordinating center. Then, height, weight, and BP were measured for each participant. Two BP readings were taken on the right arm of employees in a sitting position after resting for at least 5 min and 1-min interval by well-trained staff, with an Omron automatic digital BP monitor device (Omron HBP-1300; Omron, Kyoto, Japan) provided to all workplaces. Participants were not allowed to drink tea/coffee, smoke, and engage in any physical activity for at least 30 min prior to measurement. If the difference between the 2 measurements was greater than 5 mm Hg for systolic blood pressure (SBP) or diastolic blood pressure (DBP), a third measurement was taken and the last 2 measurements were recorded [17]. The average of two readings was used for all analyses. Height was measured without shoes using a standard right-angle device and a fixed tape measure (to the closest 0.5 cm), and body weight was measured without wearing heavy clothing using a weight measuring device (Vbody HBF-371, Omron, Kyoto, Japan).

According to the Chinese BP measurement guideline [17], HTN was defined as SBP ≥ 140 mm Hg and/or DBP ≥ 90 mm Hg, or taking antihypertensive medication, or self-reported previous medical diagnosis of HTN, which was diagnosed by CHCs or other superior medical institutions. BMI was calculated as the weight in kilograms divided by height in meters squared (kg/m^2^), and the BMI cutoff points for overweight (24∼27.9 kg/m^2^) and obesity (≥ 28 kg/m^2^) were based on the Working Group on Obesity in China guidelines [18]. According to previous report [11], smoking was defined as the use of at least 1 cigarette per day, current alcohol as consumption of at least 1 drink per week, regular exercise as more than 30 min of physical activity at least 3 times a week, stress perception as participants who reported perceiving high levels of stress, excessive intake of fatty food as participants who reported regularly overconsumption of fatty foods, and restrictive use of salt as participants who reported restriction of salty food regularly.

Employees were divided into four groups by their occupations, including manual labor workers (e.g., manufacturing, mining), desk job workers (e.g., clerical, professional technical), administrative workers (e.g., manager, director, supervisor), and others (e.g., porter, security guard). In terms of affiliated hospital [19], the workplaces were classified as those with an affiliated hospital (WAH) or without an affiliated hospital (WWH). Details have been published previously [11].

### Outcome measurements

In this post hoc analysis, we mainly conducted an exploratory analysis of the incidence of HTN, improvements of BP levels, and lifestyle factors from baseline to 24 months in the two groups. The primary outcome was 2-year incidence of HTN, defined as the proportion of employees who were free of HTN at baseline but developed HTN at 24 months. The secondary outcomes included changes of BP level and rate of smoking, drinking, regular exercise, overweight or obesity, stress perception, excessive intake of fatty food, and restrictive use of salt from baseline to 24 months.

### Statistical analysis

All employees who attended follow-up visits at 24 months and had complete primary outcome data and other variables were included in the analysis. Considering the clustering effect, a mixed effects model with the subcenters as random effect was performed to examine the intervention effect on incidence of HTN and other outcomes over time by additionally adjusting for age at recruitment (continuous), sex (male or female), marital status (married or unmarried), educational attainment (elementary or below, junior high school, college or above), employment status (manual labor workers, desk job workers, administrative workers, others), workplace-affiliated hospital (WAH, WWH), history of dyslipidemia (yes or no), history of diabetes (yes or no), history of CVD (yes or no), family history of HTN (yes or no), and pharmacological treatment (yes or no). The hierarchical statistical techniques were used to capture the heterogeneity of workplace intervention. To handle the missing data on outcome and other covariates, a multiple imputation method was employed for sensitivity analyses to robust the primary outcome analyses. Exploratory analysis was also conducted in predefined subgroups, including sex, age, marital status, educational attainment, employment status, and workplace-affiliated hospital. The results were reported as rate (%), mean, standard deviation (SD), and 95% confidence interval (CI) when appropriate. For continuous outcomes, intervention effect was defined as the difference between the 2 groups, calculated as intervention (values at 24 months minus values at baseline) minus control (values at 24 months minus values at baseline). For dichotomous outcomes, the incidence of HTN was reported by relative risk (RR) and 95% CIs compared with the control group, with the improvements of lifestyle factors by odds ratio (OR) and 95% CIs. All data analyses were conducted using R version 4.1.0. A 2-tailed *P* value less than 0.05 was considered statistically significant.

## Results

### Basic characteristics of participants at the workplaces

The participants had a mean (SD) age of 39.3 (9.1) years and were predominantly male (14,727 [60.4%]). At baseline, 10,862 participants (46.6%) had obtained a college or above education, 11,725 (48.9%) were manual labor workers, and 18,142 (74.4%) were from a workplace with an affiliated hospital. The mean SBP/DBP was 118.5/74.7 mm Hg in the entire sample. There were significant differences in age at recruitment, marital status, education attainment, employment status, workplace-affiliated hospital, the history of disease, SBP and lifestyle factors between the intervention group and the control group (Table 1). Baseline characteristics for participants who failed to be matched were shown in Table S1.

**Table 1:** Baseline characteristics for all participants who completed follow-up in the study

Variables	Intervention group (*n* = 18,170)	Control group	Total	*P* value
		**(*n* = 6,226)**	**(*n* = 24,396)**	
**Age at recruitment, mean (SD), years**	39.6 (9.1)	38.6 (9.1)	39.3 (9.1)	< 0.001
**Sex**				0.185
Male	11015 (60.6)	3712 (59.7)	14727 (60.4)	
Female	7155 (39.4)	2510 (40.3)	9665 (39.6)	
**Marital status**				< 0.001
Married	15375 (88.7)	5091 (85.4)	20466 (87.8)	
Unmarried	1965 (11.3)	870 (14.6)	2835 (12.2)	
**Education attainment**				< 0.001
Middle school or below	3978 (22.9)	1102 (18.5)	5080 (21.8)	
High school	5593 (32.3)	1767 (29.6)	7360 (31.6)	
College or above	7770 (44.8)	3092 (51.9)	10862 (46.6)	
**Employment status**				< 0.001
Manual labor worker	8992 (50.3)	2733 (44.7)	11725 (48.9)	
Desk job worker	6527 (36.5)	2447 (40.0)	8974 (37.4)	
Administrative workers	2054 (11.5)	582 (9.5)	2636 (11.0)	
Others	301 (1.7)	356 (5.8)	657 (2.7)	
**Workplace-affiliated hospital**				< 0.001
WAH	14066 (77.4)	4076 (65.5)	18142 (74.4)	
WWH	4104 (22.6)	2150 (34.5)	6254 (25.6)	
**The history of disease**				
History of dyslipidemia	1124 (6.2)	328(5.3)	1452 (6.0)	< 0.001
History of diabetes	286 (1.6)	65 (1.0)	351 (1.4)	< 0.001
History of CVD	44 (0.2)	16 (0.3)	60 (0.2)	< 0.001
Family history of hypertension	3144 (17.3)	1052 (16.9)	4196 (17.2)	< 0.001
Pharmacological treatment	294 (1.7)	106 (1.7)	400 (1.7)	0.788
**Blood pressure, mean (SD), mm Hg**				
SBP	118.6 (10.8)	118.2 (10.6)	118.5 (10.7)	0.042
DBP	74.6 (7.9)	74.8 (7.8)	74.7 (7.9)	0.306
**Lifestyle factors**				
Smoking	4629 (25.5)	1708 (27.5)	6337 (26.0)	0.003
Drinking alcohol	4365 (24.1)	1484 (23.9)	5849 (24.1)	0.732
Regular exercise	5816 (33.4)	2121 (34.3)	7937 (33.7)	0.187
Stress perception	5465 (30.2)	2104 (33.9)	7569 (31.1)	< 0.001
Overweight or obesity	7079 (39.2)	2146 (34.6)	9225 (38.0)	< 0.001
Excessive intake of fatty food	11441 (63.5)	3812 (61.4)	15253 (63.0)	0.004
Restrictive use of salt	2415 (13.3)	811 (13.0)	3226 (13.3)	0.554

Data are presented as No. (%) unless otherwise indicated

*SBP* Systolic blood pressure, *DBP* Diastolic blood pressure, *SD* Standard deviation, *WAH* Workplace with affiliated hospital, *WWH* Workplace without affiliated hospital

### Intervention effects on incidence of HTN, BP level and lifestyle factors in the intervention and control groups

**Fig. 2:**
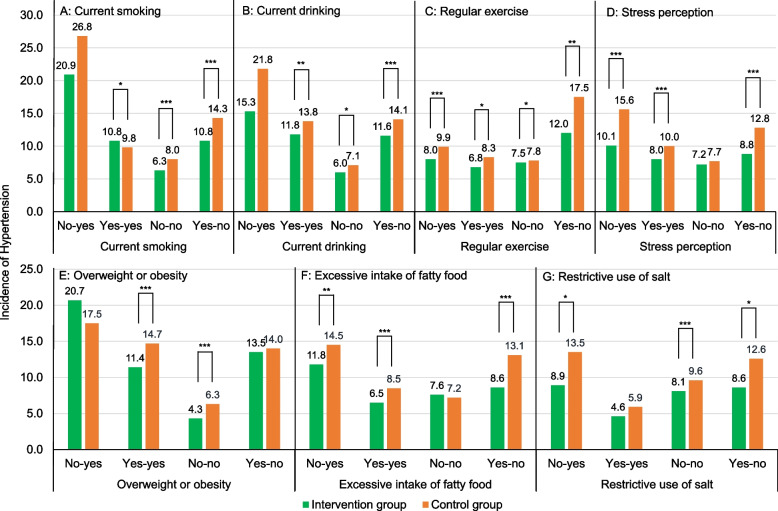
Effect of lifestyle changes on the incidence of hypertension after 2 years of intervention

Compared with the 2-year incidence of HTN of 9.6% in the control group, it was 8.0% in the intervention group, and the overall intervention effect was higher (*RR* = 0.66; 95% CI, 0.58 ~ 0.76; *P* < 0.001). The intervention effect was consistent across the subgroups examined, with administrative workers (*RR* = 0.38; 95% CI, 0.27 ~ 0.53; *P* < 0.001) and worker with history of CVD (*RR* = 0.38; 95% CI, 0.04 ~ 3.27; *P* = 0.004) had the most significant effects between the 2 groups (Table 2). Compare to people with a same or improved lifestyle before and after the intervention, those with a deteriorating lifestyle had a higher incidence of HTN (Fig. 2; Table S2).

**Table 2:** Incidence of hypertension for employees after 2 years of intervention

Variables	Change from baseline, % (95% CI)		Intervention effect	
	**Intervention group (*n* = 16,488)**	**Control group (*n* = 5,179)**	**RR (95% CI)**	***P* value**
**Overall**	1254 (8.0)	528 (9.6)	0.66 (0.58,0.76)	< 0.001
**Sex**				
Male	959 (10.2)	394 (11.9)	0.69 (0.60,0.80)	< 0.001
Female	295 (4.7)	134 (6.2)	0.52 (0.30,0.89)	0.016
**Marital status**				
Married	1027 (7.6)	411 (9.0)	0.68 (0.59,0.79)	< 0.001
Unmarried	87 (5.2)	57 (7.6)	0.53 (0.36,0.78)	0.001
**Educational attainment**				
Middle school or below	228 (6.4)	63 (6.0)	0.71 (0.45,1.10)	0.128
High school	416 (8.2)	172 (10.6)	0.63 (0.50,0.79)	< 0.001
College or above	470 (7.1)	233 (8.8)	0.57 (0.47,0.69)	< 0.001
**Employment status**				
Manual labor worker	656 (8.3)	264 (10.5)	0.56 (0.44,0.71)	< 0.001
Desk job worker	394 (7.0)	144 (6.8)	0.77 (0.62,0.96)	0.020
Administrative workers	129 (7.4)	65 (13.9)	0.38 (0.27,0.53)	< 0.001
Others	20 (7.1)	23 (7.0)	0.61 (0.25,1.51)	0.287
**Workplace-affiliated hospital**				
WAH	1011 (8.1)	349 (10.1)	0.72 (0.62,0.84)	< 0.001
WWH	243 (7.7)	179 (8.8)	0.01 (0.00,0.15)	0.001
**The history of disease**				
History of dyslipidemia	142 (13.9)	42 (14.3)	0.97 (0.64,1.48)	0.892
History of diabetes	48 (18.0)	17 (28.3)	0.54 (0.26,1.10)	0.090
History of CVD	8 (20.0)	5 (31.2)	0.38 (0.04,3.27)	0.381
Family history of hypertension	311 (11.1)	92 (10.1)	1.03 (0.76,1.39)	0.849
Pharmacological treatment	50 (18.1)	12 (12.6)	0.56 (0.44,0.71)	< 0.001
**Lifestyle factors**				
Current smoking	431 (10.8)	173 (11.1)	0.63 (0.50,0.80)	< 0.001
Current drinking	445 (11.8)	185 (14.2)	0.52 (0.41,0.65)	< 0.001
Regular exercise	422 (8.1)	208 (10.8)	0.59 (0.48,0.73)	< 0.001
Stress perception	412 (8.5)	209 (11.5)	0.55 (0.44,0.68)	< 0.001
Overweight or obesity	711 (11.6)	271 (14.2)	0.72 (0.60,0.87)	0.001
Excessive intake of fatty food	721 (7.3)	321 (9.6)	0.61 (0.51,0.72)	< 0.001
Restrictive use of salt	129 (6.2)	61 (8.5)	0.63 (0.43,0.92)	0.008

*Abbreviations*: *OR* odds ratio, *95%CI* 95% confidence interval, *WAH* workplace with an affiliated hospital, *WWH* workplace without an affiliated hospital, *CVD* cardiovascular disease. The multilevel model adjusted for age at recruitment, sex, marital status, educational attainment, employment status, workplace-affiliated hospital, history of dyslipidemia, history of diabetes, history of CVD, family history of hypertension, pharmacological treatment

After multiple imputation for missing data, the incidence of HTN in both groups was increased. The intervention effect of incidence of HTN was still significant in two groups (*RR* = 0.84, 95% CI: 0.76–0.93; *P* = 0.001), with robust effect in the other subgroups (Table S3).

Overall, the intervention effects were significant for SBP level (*β* =  − 0.7, 95% CI, − 1.06 ~  − 0.35; *P* < 0.001) and DBP level (*β* =  − 1.0, 95% CI, − 1.31 ~  − 0.76; *P* < 0.001) in two groups. Participants in the intervention group reported greater improvements in the rate of regular exercise (OR = 1.39, 95% CI, 1.28 ~ 1.50; *P* < 0.001) and excessive intake of fatty food (OR = 0.54, 95% CI, 0.50 ~ 0.59;* P* < 0.001) as well as a substantial improvement in restrictive use of salt (OR = 1.22, 95% CI, 1.09 ~ 1.36; *P* = 0.001) from baseline. Compared with those in the control group, the intervention effect of smoking, drinking, stress perception, overweight, or obesity was insignificant (Table 3). Results adjusted for different confounders are shown in Table S4. When smoking, alcohol consumption and physical activity were analyzed as continuous variables; the intervention effects of average number of cigarettes smoked per day (OR =  − 0.51, 95% CI, − 0.97 ~  − 0.05; *P* = 0.021) and weekly exercise duration (OR = 14.07, 95% CI, 8.32 ~ 19.79; *P* < 0.001) were significant and robust (Table S5). The results of comparison between groups were shown in Table S5.

**Table 3:** Changes in blood pressure level and lifestyle factors for employees in the study

**Variables**	**Intervention group (*n* = 16,488)**		**Control group (*n* = 5,179)**		**Intervention Effect**^a^	
	**Baseline, %**	**24 mo, %**	**Baseline, %**	**24 mo, %**	***β*/OR (95% CI)**	***p* value**
**SBP mean (SD), mm Hg**	118.6 (10.8)	120.1 (11.8)	118.2 (10.6)	120.5 (12.2)	− 0.7 (− 1.06, − 0.35)	< 0.001
**DBP mean (SD), mm Hg**	74.6 (7.9)	75.4 (8.7)	74.8 (7.8)	75.8 (8.6)	− 1.0 (− 1.31, − 0.76)	< 0.001
**Lifestyle factors**						
Current smoking	25.5	24.1	27.5	24.1	0.9(0.80, 1.02)	0.094
Current drinking	24.1	21.5	23.9	20.4	0.93(0.84, 1.04)	0.194
Regular exercise	33.4	45.0	34.4	39.4	1.39(1.28, 1.50)	< 0.001
Stress perception	30.2	24.0	33.9	25.6	0.93(0.85, 1.02)	0.111
Overweight or obesity	39.2	40.2	34.7	36.6	0.91(0.81, 1.01)	0.073
Excessive intake of fatty food	63.5	53.0	61.4	61.1	0.54(0.50, 0.59)	< 0.001
Restrictive use of salt	13.3	15.5	13.1	12.4	1.22(1.09, 1.36)	0.001

*OR*, odds ratio; *95%CI*, 95% confidence interval; *SBP*, systolic blood pressure; *DBP*, diastolic blood pressure; *SD*, standard deviation

^a^The multilevel model adjusted age at recruitment, sex, marital status, educational attainment, employment status, workplace-affiliated hospital, history of dyslipidemia, history of diabetes, history of CVD, family history of hypertension, pharmacological treatment

### BP intervention effects on employees with different characteristics

**Fig. 3:**
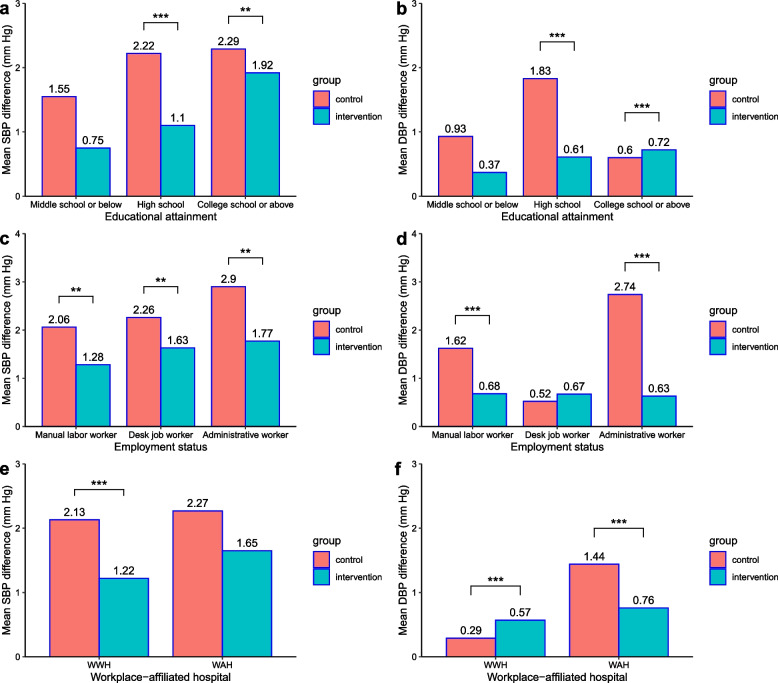
Blood pressure intervention effect on employees with different characteristics

By analyzing the BP intervention effects on employees with different characteristics, it was found that the net increase of SBP and DBP of employees with educational attainment of high school above (SBP: *β* =  − 1.38/ − 0.76, *P* < 0.05; DBP: *β* =  − 2.26/ − 0.75, *P* < 0.001), manual labor worker and administrative worker (SBP: *β* =  − 1.04/ − 1.66, *P* < 0.05; DBP: *β* =  − 1.85/ − 0.40, *P* < 0.05), and employees from a workplace with an affiliated hospital (SBP: *β* =  − 2.63, *P* < 0.001; DBP: *β* =  − 1.93, *P* < 0.001) in the intervention group was significantly lower than that in the control group (Fig. 3; Table S6).

## Discussion

In this cluster randomized controlled study with a workplace-based primary prevention interventions program of CVD, we found that a multicomponent intervention in employees with primary care in workplace significantly reduced the incidence of HTN and improved the level of BP and healthy lifestyle habits at 24 months, compared to usual care. Our study may help other workplaces with similar conditions, in domestic or foreign, to design or conduct their workplace wellness plan for improving employees’ CVD health.

Although some studies have focused on the effect of workplace health promotion on BP, few studies looked at the entire employees, let alone the incidence of HTN. We have seen a significant reduction in the incidence of HTN, which may be attributed to positive improvements in healthy lifestyles, because we found that people with deteriorating lifestyles had higher rates of developing HTN than those with an improved or same lifestyle before and after the intervention. The effects of reducing the incidence of HTN were significant across different characteristics of the occupational population and were most significant in those with a family history of CVD, which was consistent with previous findings that familial aggregation of CVD and its role as an independent risk factor for CVD is well recognized [20, 21]. The guidelines for prevention show that lifestyle interventions can reduce BP, prevent or delay the occurrence of HTN, and reduce the risk of CVD. Lifelong adherence to a healthy lifestyle is the fundamental measure for the primary prevention of CVD [6]. Practice has proved that primary prevention measures can effectively delay or avoid the occurrence of cardiovascular events, so as to reduce the incidence and mortality of CVD. Studies have shown that cardiovascular deaths in Western countries have fallen by 40% ~ 70% attributed to risk factor control [7]. Currently, there are limited studies on the impact of primary prevention on the incidence of HTN in occupational populations, some only targeted a single component [22–24], the community population rather than the occupational population [25–27], or patients with HTN rather than the whole population. Our innovative extension of multi-component primary prevention interventions to the workplaces, where workable approaches to creating a healthy environment based on the principle of adaptation to local conditions, appear to reinforce self-management behaviors and promote positive lifestyle changes.

Moreover, we saw significant differences in BP levels. Results from a meta-analysis of 147 randomized trials showed there was a 22% reduction in coronary heart disease events and a 41% reduction in stroke for a BP reduction of 10 mm Hg systolic or 5 mm Hg diastolic [28]. The Systolic Blood Pressure Intervention Trial (SPRINT) study also showed a 10% reduction in CVD risk for every 5 mm Hg drop in SBP [29]. Based on the drop of 0.7 mmHg in SBP and 1.0 mmHg in DBP in our study, the estimated reduction of CVD risk is about 10%; this implies that the difference in BP resulting from the intervention would yield significant cardiovascular risk reduction benefits. Despite the significant improvement in healthy lifestyles, SBP and DBP increased in both groups after 2 years of primary prevention interventions program compared to baseline. It is well known that BP is influenced by many factors, such as age, obesity, vascular conditions, mental status, dietary habits, and genetics [30]. At the same time, the participants of our study were healthy occupational population, rather than patients with HTN who need to lower their BP. Previous studies had also shown that there was no change in both SBP and DBP among participants in the healthy subgroup over the 6-year period [31]. Furthermore, we observed no significant improvement or even a small increase in the rate of overweight and obesity. The results of some weight loss studies showed that the enthusiasm for lifestyle changes waned over time [32, 33]. Adherence to long-term weight loss (more than 6 months) was significantly reduced, and BP increased with weight regain [34, 35]. This may explain the insignificant effect on the rate of overweight or obesity and the slight increase in BP in our study given that weight loss ultimately depends on long-term lifestyle changes [36]. This suggests that maintaining sustainable weight loss at the workplace is a key component in reducing the prevalence of overweight and obesity.

In addition, we saw the significant intervention effects on lifestyles, such as the reduced rates of excessive intake of fatty food, restrictive use of salt, and the increased rate of regular exercise. Systematic reviews of workplace interventions have shown that a multicomponent intervention would result in significant and positive changes in behaviors [37]. Our study confirmed these findings and showed greater effects than other related studies [38–41]. This may be attributed to the guideline-oriented standardized primary prevention intervention program of CVD, which is a multicomponent integrated intervention incorporating cardiovascular health education, salt restriction, proper diet, body weight control, smoking cessation, limiting alcohol consumption, physical environment promotion, physical activity, stress management, and health screening.

In our study, the intervention effect of BP was more pronounced among those with higher educational attainment. The studies found that the lower the educational attainment, the lower the level of health literacy [42–44]. Individuals with more educational attainment have higher health literacy, which was independently associated with better BP intervention effect in our study. Therefore, health education targeted at the characteristics of employees should become an important part of workplace wellness plan to comprehensively improve employees’ health literacy.

Recently published guidelines have highlighted the importance of focusing on lifestyle interventions and risk factor prevention and control [6]. Therefore, it is critical to explore and popularize feasible models in enterprises to reduce the incidence of HTN and CVD. As our study indicated, implementing a workplace-based multicomponent primary prevention interventions program for CVD may significantly reduce the incidence of HTN and improve healthy lifestyle habits. Extending this intervention to other countries or less structured workplace settings may help reduce HTN, CVD-related events, and deaths among employees, improve employees’ health, and enhance enterprise vitality.

## Limitation

This study has several limitations. First, because we did not collect data on the employers’ financial investment in the intervention, we were unable to assess the effect of money on changes in incidence of HTN between the intervention and control groups. Second, data were only collected at baseline and at the end of follow-up for the intervention and control groups, making it difficult to assess trends in outcome indicators during the intervention. Third, the extent to which the workplaces adopted the recommended interventions was not specific evaluated, even though the conditions had been improved based on the positive lifestyle changes. Fourth, this study was designed as open label, with the participants and other study investigators aware of intervention randomization. Therefore, blinded evaluation may not be feasible. We applied the outcome definition and assessment methods (the measurement of BP and definition of HTN) to minimize the subjective elements and ensure that results were as robust as possible despite the lack of blinding. In addition, significant differences of outcome variables, such as SBP, smoking, stress perception, overweight or obesity, and excessive intake of fatty food were observed between the intervention and control groups. This reminds us to be careful when interpreting and extrapolating the results. Finally, as a post hoc study of a randomized controlled trial, the statistical power could be insufficient. Since this was an exploratory research, a new trial specifically designed was needed to further answer this question.

## Conclusions

Findings of this post hoc study suggested that a workplace-based multicomponent cardiovascular primary prevention interventions program is effective and can significantly reduce the incidence of HTN and lead to positive healthy lifestyle changes. Such intervention strategy may provide a reference model for workplaces planning to implement health and wellness programs.

## Supplementary Information

**Additional file 1. **Study protocol.

**Additional file 2: Table S1.** Baseline Characteristics for Subjects Who failed to be matched in the Study. **Table S2.** Effect of lifestyle changes on the incidence of hypertension after 2 years of intervention. **Table S3.** Sensitivity Analysis for Incidence of Hypertension for Employees After Imputing the Missing Data in the Study. **Table S4.** Intervention Effect on Blood Pressure Level and Lifestyle Factors for Employees in the Study. **Table S5.** Changes in Blood Pressure Level and Lifestyle Factors for Employees in the Study. **Table S6.** Blood pressure intervention effect on employees with different characteristics.

**Additional file 3. **Checklist.

## Data Availability

The datasets used and/or analyzed during the current study are available from the corresponding author Zengwu Wang (E-mail: wangzengwu@foxmail.com) on reasonable request.

## Abbreviation

BMI: Body mass index
BP: Blood pressure
CVD: Cardiovascular disease
CDC: Chinese center for disease control and prevention
CHCs: Community health centers
CI: Confidence interval
DBP: Diastolic blood pressure
HTN: Hypertension
OR: Odds ratio
RR: Relative risk
SD: Standard deviation
SBP: Systolic blood pressure
SPRINT: Systolic Blood Pressure Intervention Trial
WAH: Workplaces with an affiliated hospital
WWH: Workplaces without an affiliated hospital
