# Supplementary material for: Workplace-based primary prevention intervention reduces incidence of hypertension: a post hoc analysis of cluster randomized controlled study
Source: BMC Med. 2023 Jun 14;21:214. doi: 10.1186/s12916-023-02915-6 (PMC10268422; doi:10.1186/s12916-023-02915-6)
Supplement: Supplementary file 1 — Additional file 1. Study protocol. [file 12916_2023_2915_MOESM1_ESM.pdf]

CONSORT

TRANSPARENT REPORTING of TRIALS

CONSORT 2010 Flow Diagram

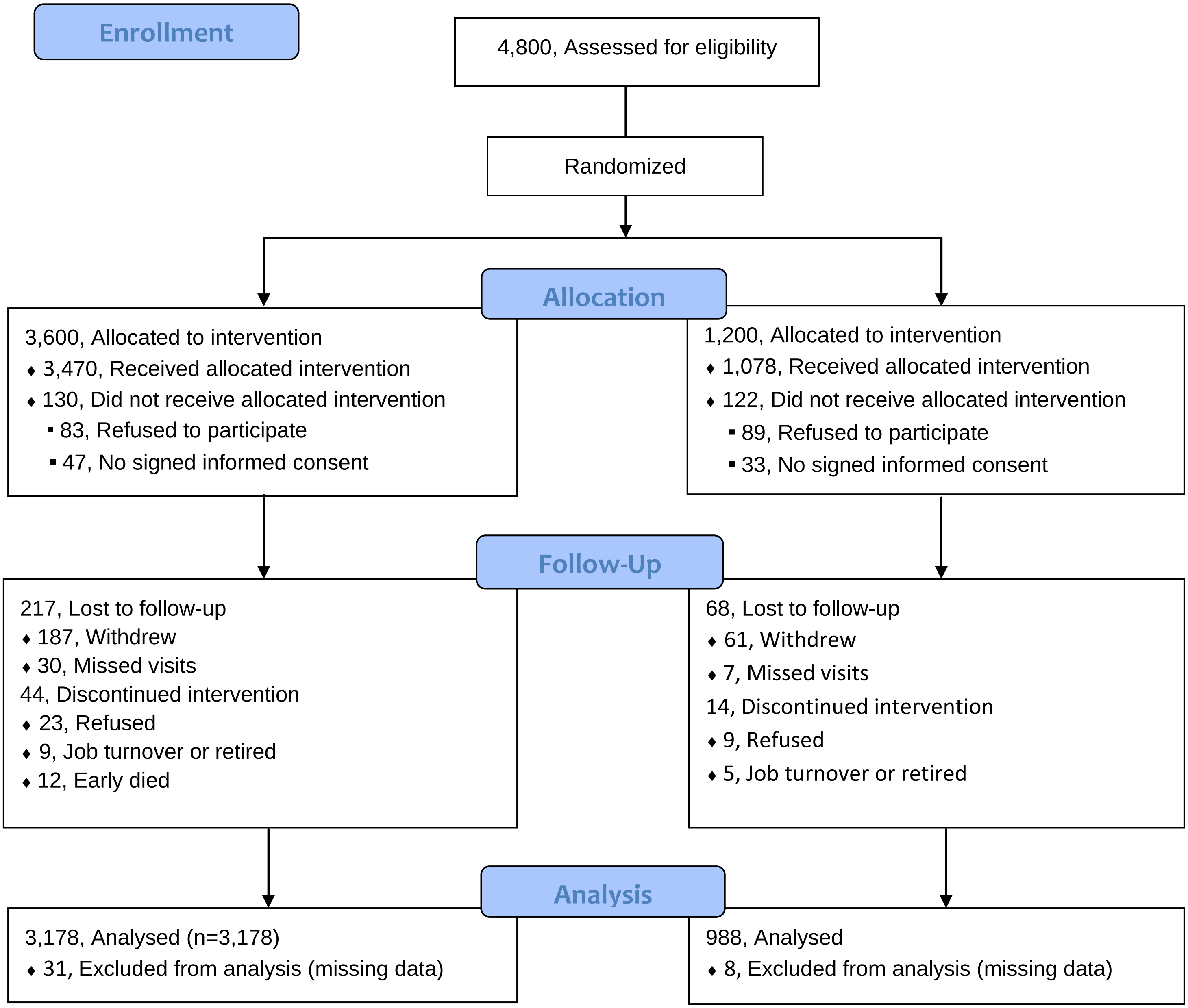

Original trial protocol and statistical analysis plan

Study title: The Standardized Management of Hypertensive Employees Program

Study Number: 2011BAI11B01

Study Phase: 2

Funding Support: Projects in the Chinese National Science & Technology Pillar Program during the 12th Five-year Plan Period

Funding Organization: Department of Science and Technology, Ministry of Science and Technology, China.

Trial Registration: ChiCTR-ECS-14004641.

Original Protocol: Version 1.0 10 September 2012

Confidentiality Statement: The information contained herein is confidential and the proprietary property of Division of Prevention and Community Health, National Center for Cardiovascular Disease, China and any unauthorized use or disclosure of such information without the prior written authorization of Division of Prevention and Community Health, National Center for Cardiovascular Disease, China is expressly prohibited.

Introduction

Raised blood pressure (BP) is established as a strong independent risk factor for cardiovascular diseases (CVD), accounting for 70% of strokes and 50% of myocardial infarction in China. On the one hand, previous investigations have reported the low level of awareness, treatment, and control of hypertension in China. However, most surveys were community based and were conducted among the general population, few reported the current prevalence and management of hypertension among the working population, and no data are available about their variations by occupation, sector, workplace ownership and medical resources. Reliable information on variations in hypertension prevalence by sex, occupation, industry classifications, etc., among Chinese working adults is crucial for developing workplace-based strategies for prevention and control of this condition.

On the other hand, from the international perspective, workplace-based intervention could be a potent approach to assist patients with uncontrolled HTN to reach BP goals.

It is well known that appropriate BP control in the hypertensive patients by lifestyle and pharmaceutical interventions can reduce CVD events effectively, while the substantial proportion remains uncontrolled. However, few studies have been specifically designed to address the HTN control among Chinese working population. Most of previous studies have been limited by small sample sizes and did not report the effectiveness of intervention on specific subgroups. Additionally, these studies did not link BP control with individual and workplace factors simultaneously.

As recommended by American Heart Association (AHA), workplace wellness programs are an important strategy to prevent major risk factors for CVD. Therefore, we will conducted a standardized management of hypertensive employees' program, aiming to assess the prevalence, awareness, treatment, and control of hypertension among the Chinese working population, and to develop a multicomponent intervention strategy that combined workplace health promotion and standardized management of hypertensive employees to improve BP control, based on the baseline survey. Herein, we hypothesize that this intervention program could improve BP control in workplaces across China, and a cluster-controlled trial will be conducted to test this hypothesis.

Primary Objective: Assess the change in the percentage of patients with controlled BP from baseline to the 24-month follow-up.

Secondary Objective(s):

- Assess the changes of BP level from baseline to the 24-month follow-up.
- Assess the changes in prevalence of smoking, drinking, exercise, and overweight/obese, from baseline to the 24-month follow-up.

Exploratory Objective(s):

- Assess the intervention effect across the subgroups of education, occupation status, workplace ownership, and workplace-affiliated hospital.

Study design

The study is a two-phase design. First, a cross-sectional survey designed to evaluate the prevalence and management of hypertension among Chinese working population. Then, a cluster-controlled trial conducted to test the effect of a multicomponent intervention strategy on improving hypertension control in workplaces.

For this program, a coordinating center will be established at Fuwai Hospital, Beijing. Then, 20 urban medical institutions will be selected as sub-centers, representing various geographic locations in China. Subsequently, 2 to 4 workplaces will be recruited by each sub-center, to test the feasibility of the protocol in work settings, all of which should meet the following criteria: (1) more than 50 patients can be recruited per site; (2) workplaces within the same sub-center should be matched by sector, ownership, size, economic level and medical condition; (3) none similar programs were conducted before; (4) non-medical institutions; (5) agrees to participate in the program for a minimum of 2 years; (6) agrees to arrange at least one full-time or part-time project officer to execute the workplace wellness program; and (7) can select a designated hospital clinic for implementing the standardized HTN management for 2 years.

Within each sub-center, 1 to 3 workplaces will be assigned to the intervention group, and one workplace assigned to the control group, with approval from manager of each workplace. To minimize the potential imbalance between groups, workplaces assigned to the intervention and control group within each sub-center, should be matched by sector, ownership, size, economic level and medical condition. Within each sub-center, at least 50 potential participants will be selected and invited for

baseline assessments at each workplace, and the patients will be allocated to intervention or usual care in a 3:1 ratio.

For the intervention patients alone, there will be monthly follow-up visits within the 2 years duration by their physicians. Patients in the control group carry on with their usual health care.

Study Duration:

Subjects will receive intervention for a minimum of 24 months.

Study Inclusion and Exclusion Criteria:

Inclusion Criteria:

1. Subject has a clinical diagnosis of HTN;
2. Subject is aged 18 to 60 years;
3. Subject is under a fixed term employment contract;
4. Subject has provided signed consent form;
5. Subject agrees to participate in the study for the full 2 years duration.

Exclusion criteria

1. Subject has secondary hypertension;
2. Subject has acute myocardial infarction or stroke less than 3 months;
3. Women who self-reports being pregnant or within 3 months postpartum;
4. Subject with life expectancy of less than 2 years;
5. Subject with psychiatric illness, hearing difficulty, or physical incapacitation;

6. Subject is a healthcare provider.

Intervention Strategy

The intervention will last for 2 years, and include two components: (1) workplace wellness program for primary prevention of CVD, the development of comprehensive health promotion within workplace to increase individual employees' awareness of risk factors and produce sustained behavior change, and (2) a standardized protocol of HTN management, the implementation of guideline-based HTN management by patients' clinical teams to improve HTN control.

1. Workplace component

A comprehensive, culturally sensitive workplace wellness program aiming at improving employees' cardiovascular health will be implemented, in reference to the recommendations from AHA, and Chinese Guidelines for the Management of Hypertension. The program should better be integrated into the organizational structure of the workplace, and new initiatives should rely on existing valid sources. The project officers will be responsible for coordinating all events within workplaces. It should be noted that the program will be delivered to the whole workforce population, despite only certain number of hypertensive patients will be selected for assessments on each site. Specifically, the workplace wellness program includes the following components: (1) CVD health education. The project officers should organize lectures, put up posters and/or send text messages to educate employees

about the knowledge of risk or prevention of CVD. The topics including prevalence, risk factors and management of CVD, and strategies for behavioral changes, etc. (2) Reasonable diet. Nutrition education and/or healthy diets information were provided to the employees. In addition to the previously-served food, easy access, affordable, healthy foods (e.g., low-salt, low-fat food) were offered in the cafeteria, and the participants were encouraged to select it to eat from the menu when they have lunch in the cafeteria in their worksites. (3) Tobacco cessation. Tobacco control regulation was developed to improve tobacco cessation among the employees, and no smoking was permitted in the workplaces. (4) Environment promotion. The improvements of physical environment include modifying workstations and office layouts to decrease sedentary behavior, encouraging physical activity. (5) Physical activity. Participants were encouraged to increasing physical activity within exercise breaks during working hours. Accessible indoor or outdoor sports facilities were provided to engage employees in regular physical activity, including an indoor walking path. (6) Stress management. Relaxation techniques were provided by the employees specialized in meditation, tai-chi or deep breathing for dealing with stress monthly. (7) Health screening. Health checkup were offered annually, and feedback was provided to identify the key risk factors.

2. Clinic component

Employees with hypertension will be managed for at least 2 years by physicians in the clinics trained by a standardized BP management protocol. The protocol, which is based on Chinese Guidelines for the Management of Hypertension, include the

following: classification of HTN, stratification of global risk, targets for BP control, the principles of treatment, lifestyle changes, drug treatment, and follow-up management. BP control targets are as follows: <140/90 mmHg for uncomplicated hypertension; <130/80 mmHg for patients with diabetes, cerebrovascular disease, stable coronary heart disease, or chronic renal failure. The principles of treatment is based on patients' risk stratification. The physicians should make oral health education on lifestyle changes and prescribe the drugs according to patients' conditions. The patients will be instructed to visit the designated clinics monthly until the end of this trail, during which the measurement of BP as well as the adjustment of individual therapeutic regimen made. The project officers should be responsible for supervising and assisting hypertensive patients in regular follow-up. Moreover, patients will be referred to a higher-level hospital when necessary, and then return to the designated clinics for follow-up when they are in a stable condition.

Effectiveness assessments

There will be two examinations for both groups, at baseline and 24 months (after 2 years intervention). Indicators including BP, BMI and lifestyle factors will be assessed.

All outcome assessments will be conducted at baseline and at 24-month follow-up in exactly the same way in the clinics for all patients, irrespective of their assignment to intervention or control group. For the intervention patients, monthly follow-up visits will be made within the 2 years duration to document the BP and lifestyle factors.

Statistical Methods:

All patients who attended follow-up visits at 24 months and had complete data for the primary outcome and other variables were included in the analysis. The baseline differences between patients included and unincluded, patients in the intervention group and those in the control group, will be examined by mixed-effects model. All patients who attend follow-up visits at 24 months and have complete data for the primary outcome may be included in the analysis, without imputation for missing data. The intervention effect will be performed as the difference between two groups, it will be calculated as intervention (values at 24 months minus values at baseline) minus control (values at 24 months minus values at baseline), and evaluated by mixed-effects models (PROC MIXED for continuous variables, PROC GLIMMIX for categorical variables), which included a random cluster effect (workplace). Group (intervention, control), all the baseline covariates, and potential interaction terms were used for adjustment in all models. The subgroup analyses of primary outcome will be conducted to assess the intervention effect on subgroups of education, occupation status, workplace ownership, and workplace-affiliated hospital. To evaluate the robustness of the results of the primary analyses, multiple-imputation method was adopted to handle missing data on outcome and covariates in a sensitivity analysis. The results will be summarized as rate (%), mean, standard deviation (SD), standard error (SE), and 95% confidence interval (CI) when appropriate. All data analyses will be conducted using SAS 9.4 (SAS institute, Cary, NC, USA) and a two-sided *P*-value

<0.05 will be considered statistically significant.

Sample Size Considerations:

The study is designed as an effectiveness trial of active interventions, compared with the usual care group. On the basis of previous literature, we expected that 20% of patients in the control group would be properly controlled, whereas we expected patients in the intervention group to increase this to 50% control at 24-month follow-up. Considering the intervention benefits and ethical issues, the study was designed to recruit patients using a 3:1 ratio. Cluster randomization power analysis was performed for the two independent proportions, and with a 2.5% type I error and intra-cluster correlation coefficient (ICC) of 0.01, we estimated that a sample size of 3,000 intervention patients (cluster size 75) and 1,000 control patients (cluster size 50) would provide 90% power to detect an improvement of 25% in BP control between the two groups and allowing 20% loss to follow-up in each group. To ensure an enough power, 3,600 intervention patients and 1,200 control patients were recruited in this study.

References

1. National Center for Cardiovascular Disease, China. Report on cardiovascular diseases in china (2016). Beijing: Encyclopedia of China Publishing House; 2017.
2. Wang Z, Chen Z, Zhang L, Wang X, Hao G, Zhang Z, et al. Status of hypertension in China: Results from the China hypertension survey, 2012-2015 [published online February 15, 2018]. Circulation.doi: 10.1161/CIRCULATIONAHA.117.032380.
3. Thomopoulos C, Parati G, Zanchetti A. Effects of blood pressure lowering on outcome incidence in hypertension. 1. Overview, meta-analyses, and meta-regression

analyses of randomized trials. J Hypertens. 2014;32:2285-95.

4. Shen Y, Wang X, Wang Z, Zhang L, Chen Z, Zhu M, et al. Prevalence, awareness, treatment, and control of hypertension among chinese working population: Results of a workplace-based study. J Am Soc Hypertens. 2018;12:311-322.

5. Watson AJ, Singh K, Myint UK, Grant RW, Jethwani K, Murachver E, et al. Evaluating a web-based self-management program for employees with hypertension and prehypertension: A randomized clinical trial. Am Heart J. 2012;164:625-631

6. Eng JY, Moy FM, Bulgiba A. Impact of a workplace health promotion program on employees' blood pressure in a public university. PLoS One. 2016;11:e0148307

7. Lowensteyn I, Berberian V, Belisle P, DaCosta D, Joseph L, Grover SA. The measurable benefits of a workplace wellness program in Canada: Results after one year. J Occup Environ Med. 2017

8. Liu L, Li M, Song S, Shi A, Cheng S, Dang X, et al. Effects of long-term psychological intervention on blood pressure and health-related quality of life in patients with hypertension among the Chinese working population. Hypertens Res. 2017;40:999-1007

9. Carnethon M, Whitsel LP, Franklin BA, Kris-Etherton P, Milani R, Pratt CA, et al. Worksite wellness programs for cardiovascular disease prevention: A policy statement from the American Heart Association. Circulation. 2009;120:1725-1741

10. Writing group of 2010 Chinese guidelines for the management of hypertension. 2010 Chinese guidelines for the management of hypertension. Chin J Hypertens. 2015;23:24-43

11. Zhou BF. Effect of body mass index on all-cause mortality and incidence of cardiovascular diseases--report for meta-analysis of prospective studies open optimal cut-off points of body mass index in chinese adults. Biomed Environ Sci. 2002;15:245-252

12. Wang Z, Zhang L, Chen Z, Wang X, Shao L, Guo M, et al. Survey on prevalence of hypertension in China: Background, aim, method and design. Int J Cardiol. 2014;174:721-723

13. Xu T, Wang Y, Li W, Chen WW, Zhu M, Hu B, et al. Survey of prevalence, awareness, treatment, and control of hypertension among Chinese governmental and institutional employees in Beijing. Clin Cardiol. 2010;33:E66-72

14. He FJ, Wu Y, Feng XX, Ma J, Ma Y, Wang H, et al. School based education programme to reduce salt intake in children and their families (school-edusalt): Cluster randomised controlled trial. BMJ. 2015;350:h7

Appendix A: Informed consent

You are participating in a workplace-based multicomponent intervention program that combined workplace health promotion and standardized management of hypertension. The program is organized by the Fuwai Hospital, Peking Union Medical College &

Chinese Academy of Medical Sciences (National Center for Cardiovascular Disease, China), and conducted by your workplace and local medical institutions. The program will select the project officer in your workplace, who will be responsible for conducting health education and lifestyle guidance for the hypertensive patients in workplaces, supervising and assisting them in regular follow-up at the designated hospital, to better improve their blood pressure control, reduce the occurrence of cardiovascular diseases, and improve the quality of life.

If you are to be participated in this program, for the subsequent 2 years, the project officer in your workplace will be responsible for guidance of your lifestyle modification, and assist doctors in designated hospital for standardization management of your blood pressure. During the follow-up management, you need to cooperate in the following aspects: participate in the health promotion activities of the workplace actively; visit your designated doctor regularly so that the doctor can observe the changes and the intervention effect over time; the doctor is obligated to explain the hypertension related knowledge and answer your questions during your follow-up visits. According to the requirements of this program, at the beginning of the program and two years later at the end of this program, you should have the detections of fasting glucose, total cholesterol, triglycerides, high-density lipoprotein cholesterol and electrocardiogram, all of these detections are free of charge. The drugs that the doctor chooses for you in the course of treatment are all conventional drugs, and there are no special medication requirements in this program.

Participation in the program is voluntary. You have the right to withdraw from

this activity at any time.

Before signing this consent form, I have read or others have read to me, and I have the opportunity to inquire and received a satisfactory reply. I will volunteer for this program. After participating in the program, I am willing to accept the follow-up visits and hypertension management by the doctor in designated hospital and project officer in workplace.

Subjects with signature________________ Date_________________

I assure you that I have introduced my work to the patient.

Doctor with signature________________ Date_________________

Appendix B: Questionnaire (For Baseline and 24 month)

Sub-center ID _______ No. of work unit _______ No. of employee _______

Section A: Basic information

A1. Name: _______

A2. Gender: ①Male ②Female

A3. Race: _______

A4. Occupation: ①Managerial ②Professional technical ③Clerical

④Sales and services ⑤Workers ⑥Others_______

A5. Date of birth: Year_______ Month ______ Day ______

Section B: Health status (Determined according to the current Chinese clinical

guidelines)

B1. Do you have hypertension? ①No ②Yes ③Unclear

If yes, the duration is: ______years; Current BP levels (SBP/DBP): ______/______mmHg

Do you take the BP-lowering drugs currently? ①No ②Yes

B2. Do you have dyslipidemia? ①No ②Yes ③Unclear

If yes, the duration is: ______years; Current total cholesterol (TC): ______ mmol/L

or ______ mg/dL

Do you take the TC-lowering drugs currently? ①No ②Yes

B3. Do you have diabetes? ①No ②Yes ③Unclear

If yes, the duration is: ______years; Current fasting blood-glucose (FBG): ______ mmol/L or ______ mg/dL

Do you take the FBG-lowering drugs currently? ①No ②Yes

B4. Do you have myocardial infarction? ①No ②Yes ③Unclear

If yes, the duration is: ______years.

B5. Do you have stroke? ①No ②Yes ③Unclear

If yes, the duration is: ______years.

Section C: Family history (Positive is defined as parents/siblings suffer from the diseases before the age of 50 years)

C1. Hypertension ①No ②Yes ③Unclear

C2. Stroke ①No ②Yes ③Unclear

C3. Coronary heart disease ①No ②Yes ③Unclear

C4. Diabetes ①No ②Yes ③Unclear

Section D: Lifestyle factors

D1. Do you prefer eating salty food regularly? ①No ②Yes

D2. Do you perceive high levels of stress recently? ①No ②Yes

D3. Do you smoke currently (at least one cigarette per day)? ①No ②Yes

If yes, how many cigarettes do you smoke daily on average? ______

D4. Do you drink currently (consumption of at least 1 alcoholic beverage per week)?

①No ②Yes

If yes, how many times do you drink per week on average? ______

Section E: Physical examination and Biochemical test

E1. Height: ______ cm; Weight: ______ kg

E2. Blood pressure (SBP/DBP):

The first time ___/___ mmHg; the second time ___/___ mmHg;

E3: Fasting glucose: ______ mmol/L or ______ mg/dL

E4: Triglycerides: ______ mmol/L or ______ mg/dL

E5: Total cholesterol: ______ mmol/L or ______ mg/dL

E6: High density lipoprotein cholesterol: ______ mmol/L or ______ mg/dL

Appendix C: Questionnaire (For monthly follow-up in intervention group)

Sub-center ID _______ No. of work unit _______ No. of employee _______

Section A: Physical examination

A1. Blood pressure (SBP/DBP):

The first time ___/___ mmHg; the second time ___/___ mmHg;

Section B: Lifestyle factors

B1. Do you prefer eating salty food regularly? ①No ②Yes

B2. Do you perceive high levels of stress recently? ①No ②Yes

B3. Do you smoke currently (at least one cigarette per day)? ①No ②Yes

If yes, how many cigarettes do you smoke daily on average? ______

B4. Do you drink currently (consumption of at least 1 alcoholic beverage per week)?

①No ②Yes

If yes, how many times do you drink per week on average? ______
