# Supplementary material for: Workplace-based primary prevention intervention reduces incidence of hypertension: a post hoc analysis of cluster randomized controlled study
Source: BMC Med. 2023 Jun 14;21:214. doi: 10.1186/s12916-023-02915-6 (PMC10268422; doi:10.1186/s12916-023-02915-6)
Supplement: Supplementary file 2 — Additional file 2: Table S1. Baseline Characteristics for Subjects Who failed to be matched in the Study. Table S2. Effect of lifestyle changes on the incidence of hypertension after 2 years of intervention. Table S3. Sensitivity Analysis for Incidence of Hypertension for Employees After Imputing the Missing Data in the Study. Table S4. Intervention Effect on Blood Pressure Level and Lifestyle Factors for Employees in the Study. Table S5. Changes in Blood Pressure Level and Lifestyle Factors for Employees in the Study. Table S6. Blood pressure intervention effect on employees with different characteristics. [file 12916_2023_2915_MOESM2_ESM.docx]

| **Table S1. Baseline Characteristics for Subjects Who failed to be matched in the Study** | | | | | |
| --- | --- | --- | --- | --- | --- |
| **Variables** | | **Intervention group (n=4,740)** | **Control group**  **(n=1.281)** | **Total**  **(n= 6,021)** | ***P* value** |
| **Age at recruitment, mean (SD), y** |  | 40.96 (11.07) | 40.10 (9.77) | 40.78 (10.82) | 0.012 |
| **Sex** |  |  |  |  | 0.710 |
| Male |  | 2991 (63.1) | 800 (62.5) | 3791 (63.0) |  |
| Female |  | 1748 (36.9) | 480 (37.5) | 2228 (37.0) |  |
| **Marital status** |  |  |  |  | 0.246 |
| Married |  | 3442 (84.7) | 826 (83.1) | 4268 (84.3) |  |
| Unmarried |  | 624 (15.3) | 168 (16.9) | 792 (15.7) |  |
| **Education attainment** |  |  |  |  | <0.001 |
| Middle school or below |  | 1336 (32.9) | 106 (10.7) | 1442 (28.5) |  |
| High school |  | 1401 (34.5) | 309 (31.1) | 1710 (33.8) |  |
| College or above |  | 1328 (32.7) | 579 (58.2) | 1907 (37.7) |  |
| **Employment status** |  |  |  |  | <0.001 |
| Manual labor worker |  | 2592 (55.6) | 509 (40.7) | 3101 (52.5) |  |
| Desk job worker |  | 1363 (29.2) | 389 (31.1) | 1752 (29.6) |  |
| Administrative workers |  | 381 (8.2) | 184 (14.7) | 565 (9.6) |  |
| Others |  | 324 (7.0) | 170 (13.6) | 494 (8.4) |  |
| **Workplace-affiliated hospital** |  |  |  |  | <0.001 |
| WAH |  | 2416 (53.5) | 822 (64.2) | 3238 (55.9) |  |
| WWH | | 2101 (46.5) | 458 (35.8) | 2559 (44.1) |  |
| **The history of disease** | |  |  |  |  |
| History of dyslipidemia | | 291 (6.2) | 107 (8.4) | 398 (6.6) | <0.001 |
| History of diabetes | | 105 (2.2) | 24 (1.9) | 129 (2.2) | <0.001 |
| History of CVD | | 24 (0.5) | 2 (0.2) | 26 (0.4) | <0.001 |
| Family history of hypertension | | 726 (15.4) | 301 (23.5) | 1027 (17.1) | <0.001 |
| Pharmacological treatment | | 143 (3.6) | 25 (2.1) | 168 (3.3) | 0.018 |
| **Blood pressure,** **mean (SD), mm Hg** | | | |  |  |
| SBP | | 124.55 (15.62) | 122.65 (15.92) | 124.15 (15.70) | <0.001 |
| DBP | | 78.38 (11.09) | 77.81 (11.04) | 78.26 (11.08) | 0.108 |
| **Lifestyle factors** | | |  |  |  |
| Smoking | | 1287 (27.4) | 364 (28.5) | 1651 (27.6) | 0.458 |
| Drinking alcohol | | 990 (21.1) | 322 (25.3) | 1312 (22.0) | 0.002 |
| Regular exercise | | 1655 (37.5) | 446 (35.4) | 2101 (37.1) | 0.180 |
| Stress perception | | 1070 (22.7) | 477 (37.4) | 1547 (25.8) | <0.001 |
| Overweight or obesity | | 1853 (39.3) | 466 (36.9) | 2319 (38.8) | 0.129 |
| Excessive intake of fatty food | | 2795 (60.0) | 699 (54.7) | 3494 (58.9) | 0.001 |
| Restrictive use of salt | | 805 (17.1) | 198 (15.5) | 1003 (16.8) | 0.173 |

Subjects Who failed to be matched included 3,147 employees who lost to follow-up and 2,874 with incomplete information. Data are presented as No. (%) unless otherwise indicated. SBP, systolic blood pressure; DBP, diastolic blood pressure; SD: standard deviation; WAH, workplace with affiliated hospital; WWH, workplace without affiliated hospital.

| **Table S2. Effect of lifestyle changes on the incidence of hypertension after 2 years of intervention** | | | | |
| --- | --- | --- | --- | --- |
| **Variables** | **Change From Baseline, % (95% CI)** | | **Intervention Effect** | |
|  | **Intervention group (n=16,488)** | **Control group (n=5,179)** | **RR (95% CI)** | ***P* value** |
| Current smoking |  |  |  |  |
| No-yes | 121(20.9) | 45(26.8) | 0.76(0.45,1.27) | 0.297 |
| Yes-yes | 343(10.8) | 114(9.8) | 0.75(0.57,1.00) | 0.047 |
| No-no | 693(6.3) | 299(8.0) | 0.67(0.57,0.80) | <0.001 |
| Yes- no | 87(10.8) | 55(14.3) | 0.38(0.23,0.63) | <0.001 |
| Current drinking |  |  |  |  |
| No-yes | 133(15.3) | 53(21.8) | 0.68(0.43,1.09) | 0.108 |
| Yes-yes | 294(11.8) | 117(13.8) | 0.61(0.46,0.82) | 0.001 |
| No-no | 659(6.0) | 277(7.1) | 0.79(0.66,0.94) | 0.010 |
| Yes- no | 147(11.6) | 64(14.1) | 0.40(0.26,0.61) | <0.001 |
| Regular exercise |  |  |  |  |
| No-yes | 247(8.0) | 71(9.9) | 0.55(0.39,0.76) | <0.001 |
| Yes-yes | 276(6.8) | 121(8.3) | 0.72(0.56,0.93) | 0.013 |
| No-no | 509(7.5) | 213(7.8) | 0.76(0.61,0.94) | 0.011 |
| Yes- no | 133(12.0) | 78(17.5) | 0.51(0.35,0.75) | 0.001 |
| Stress perception |  |  |  |  |
| No-yes | 158(10.1) | 69(15.6) | 0.48(0.32,0.71) | <0.001 |
| Yes-yes | 183(8.0) | 87(10.0) | 0.55(0.41,0.75) | <0.001 |
| No-no | 667(7.2) | 245(7.7) | 0.83(0.69,1.01) | 0.061 |
| Yes- no | 225(8.8) | 121(12.8) | 0.57(0.44,0.75) | <0.001 |
| Overweight or obesity |  |  |  |  |
| No-yes | 165(20.7) | 68(17.5) | 0.65(0.42,1.00) | 0.050 |
| Yes-yes | 580(11.4) | 192(14.7) | 0.65(0.53,0.79) | <0.001 |
| No-no | 348(4.3) | 167(6.3) | 0.56(0.44,0.70) | <0.001 |
| Yes- no | 95(13.5) | 36(14.0) | 1.19(0.67,2.11) | 0.554 |
| Excessive intake of fatty food | |  |  |  |
| No-yes | 216(11.8) | 99(14.5) | 0.67(0.49,0.90) | 0.008 |
| Yes-yes | 401(6.5) | 226(8.5) | 0.67(0.55,0.81) | <0.001 |
| No-no | 295(7.6) | 103(7.2) | 0.78(0.58,1.05) | 0.105 |
| Yes- no | 316(8.6) | 92(13.1) | 0.42(0.31,0.58) | <0.001 |
| Restrictive use of salt |  |  |  |  |
| No-yes | 111(8.9) | 31(13.5) | 0.53(0.32,0.87) | 0.011 |
| Yes-yes | 57(4.6) | 26(5.9) | 0.74(0.42,1.30) | 0.294 |
| No-no | 1000(8.1) | 435(9.6) | 0.69(0.60,0.80) | <0.001 |
| Yes- no | 72(8.6) | 35(12.6) | 0.52(0.31,0.88) | 0.015 |

Abbreviations: RR, relative risk; 95%CI, 95% confidence interval. The multilevel model adjusted for age at recruitment, sex, marital status, educational attainment, employment status, workplace-affiliated hospital, history of dyslipidemia, history of diabetes, history of CVD, family history of hypertension, pharmacological treatment. No-yes: Adopting unhealthy lifestyle before intervention and improving to healthy lifestyle after intervention. Yes-yes: Adopting unhealthy lifestyle before and after intervention.

No-no: Adopting healthy lifestyle before and after intervention. Yes-no: Adopting healthy lifestyle before intervention but deteriorating to unhealthy lifestyle after intervention.

| **Table S3. Sensitivity Analysis for Incidence of Hypertension for Employees After Imputing the Missing Data in the Study** | | | | |
| --- | --- | --- | --- | --- |
| **Variables** | **Change From Baseline, % (95% CI)** | | **Intervention Effect** | |
|  | **Intervention group (n=16,488)** | **Control group (n=5,179)** | **RR (95% CI)** | ***P* value** |
| **Overall** | 2325(12.8) | 835(13.4) | 0.84(0.76, 0.93) | 0.001 |
| **Sex** |  |  |  |  |
| Male | 1669(15.2) | 575(15.5) | 0.83(0.73, 0.94) | 0.003 |
| Female | 656(9.2) | 260(10.4) | 0.83(0.69, 1.00) | 0.048 |
| **Marital status** |  |  |  |  |
| Married | 2017(12.8) | 685(13.1) | 0.88(0.79, 0.98) | 0.023 |
| Unmarried | 308(12.9) | 150(15.1) | 0.59(0.42, 0.82) | 0.002 |
| **Educational attainment** |  |  |  |  |
| Middle school or below | 616(14.3) | 160(13.0) | 0.84(0.62, 1.14) | 0.259 |
| High school | 647(11.4) | 239(13.3) | 0.76(0.63, 0.93) | 0.006 |
| College or above | 1062(13.0) | 436(13.6) | 0.71(0.62, 0.82) | <0.001 |
| **Employment status** |  |  |  |  |
| Manual labor worker | 1246(13.6) | 403(14.4) | 0.88(0.73, 1.06) | 0.180 |
| Desk job worker | 761(11.6) | 263(10.7) | 0.81(0.68, 0.95) | 0.010 |
| Administrative workers | 290(13.7) | 132(21.7) | 0.52(0.41, 0.67) | <0.001 |
| Others | 28(9.3) | 37(10.4) | 0.34(0.18, 0.64) | 0.001 |
| **Workplace-affiliated hospital** |  |  |  |  |
| WAH | 685(16.7) | 250(11.6) | 0.01(0.00, 0.09) | <0.001 |
| WWH | 1640(11.7) | 585(14.4) | 0.80(0.72, 0.90) | <0.001 |
| **The history of disease** |  |  |  |  |
| History of dyslipidemia | 188(16.7) | 56(17.0) | 0.91(0.64, 1.29) | 0.607 |
| History of diabetes | 59(20.6) | 18(27.7) | 0.63(0.33, 1.19) | 0.153 |
| History of CVD | 9(20.5) | 5(31.2) | 0.38(0.06, 2.69) | 0.336 |
| Family history of hypertension | 453(14.4) | 143(13.6) | 1.01(0.79, 1.28) | 0.953 |
| Pharmacological treatment | 58(19.5) | 17(15.7) | 0.88(0.73, 1.06) | 0.180 |
| **Lifestyle factors** |  |  |  |  |
| Current smoking | 743(16.0) | 242(14.2) | 0.89(0.74, 1.08) | 0.230 |
| Current drinking | 718(16.3) | 272(18.2) | 0.62(0.51, 0.75) | <0.001 |
| Regular exercise | 725(12.0) | 303(14.2) | 0.74(0.63, 0.88) | <0.001 |
| Stress perception | 669(12.2) | 332(15.7) | 0.62(0.52, 0.74) | <0.001 |
| Overweight or obesity | 1161(16.3) | 374(17.3) | 0.90(0.78, 1.05) | 0.173 |
| Excessive intake of fatty food | 1438(12.5) | 508(13.3) | 0.76(0.67, 0.87) | <0.001 |
| Restrictive use of salt | 271(11.2) | 99(12.2) | 0.79(0.60, 1.05) | 0.104 |

Abbreviations: OR, relative risk; 95%CI, 95% confidence interval; WAH, workplace with an affiliated hospital; WWH, workplace without an affiliated hospital; CVD, cardiovascular disease. The multilevel model adjusted for age at recruitment, sex, marital status, educational attainment, employment status, workplace-affiliated hospital, history of dyslipidemia, history of diabetes, history of CVD, family history of hypertension, pharmacological treatment. Increase, same and decrease refers to the increase/same/decrease in the rate of lifestyle adoption after 2 years compared to baseline.

| **Table S4. Intervention Effect on Blood Pressure Level and Lifestyle Factors for Employees in the Study** | | | |
| --- | --- | --- | --- |
| **Variables** | **Intervention Effect (Model 1)** | **Intervention Effect (Model 2)** | **Intervention Effect (Model 3)** |
|  | **β/OR (95% CI)** | **β/OR (95% CI)** | **β/OR^d^ (95% CI)** |
| **SBP mean (SD), mm Hg** | -0.63(-0.98, -0.28) | -0.66(-1.01, -0.31) | -0.76(-1.11, -0.4) |
| **DBP mean (SD), mm Hg** | -1.02(-1.29, -0.74) | -1.00(-1.27, -0.73) | -1.08(-1.36, -0.80) |
| **Lifestyle factors** |  |  |  |
| Current smoking | 0.92(0.82, 1.03) | 0.88(0.78, 0.99) | 0.91(0.81, 1.03) |
| Current drinking | 0.95(0.86, 1.06) | 0.92(0.83, 1.02) | 0.94(0.84, 1.04) |
| Regular exercise | 1.48(1.37, 1.61) | 1.47(1.36, 1.59) | 1.40(1.29, 1.51) |
| Stress perception | 1.04(0.95, 1.13) | 1.04(0.96, 1.14) | 0.95(0.88, 1.04) |
| Overweight or obesity | 0.91(0.82, 1.01) | 0.91(0.82, 1.01) | 0.91(0.82, 1.01) |
| Excessive intake of fatty food | 0.52(0.48, 0.56) | 0.53(0.49, 0.57) | 0.54(0.50, 0.59) |
| Restrictive use of salt | 1.27(1.14, 1.42) | 1.27(1.14, 1.41) | 1.23(1.10, 1.37) |

Model 1: unadjusted; Model 2: adjusted sex, age; Model 3: adjusted sex, age, marital status, education attainment, employment status, workplace-affiliated hospital; OR, odds ratio; 95%CI, 95% confidence interval; SBP, systolic blood pressure; DBP, diastolic blood pressure; SD: standard deviation. ^d^ β represents the intervention effect of SBP and DBP, OR represents the intervention effect of lifestyle factors.

| **Table S5. Changes in Blood Pressure Level and Lifestyle Factors for Employees in the Study** | | | | | | | | | | | |
| --- | --- | --- | --- | --- | --- | --- | --- | --- | --- | --- | --- |
| **Variables** | **Intervention group (n=16,488)** | | | | **Control group (n=5,179)** | | | | **Intervention Effect^a^** | | |
|  | **Baseline, %** | **24 mo, %** | **Change From Baseline, % (95% CI)** | **Baseline, %** | | **24 mo, %** | **Change From Baseline, % (95% CI)** | **β/OR (95% CI)** | | ***p* value** |  |
| **SBP mean (SD), mm Hg** | 118.6(10.8) | 120.1(11.8) | 1.6(1.4, 1.7)^b^ | 118.2(10.6) | | 120.5(12.2) | 2.2(1.9, 2.5)^b^ | -0.7(-1.06, -0.35) | | <0.001 |  |
| **DBP mean (SD), mm Hg** | 74.6(7.9) | 75.4(8.7) | 0.7(0.6, 0.8)^b^ | 74.8(7.8) | | 75.8(8.6) | 1.0(0.8, 1.3)^b^ | -1.0(-1.31, -0.76) | | <0.001 |  |
| **Lifestyle factors (qualitative data)** |  |  |  |  | |  |  |  | |  |  |
| Current smoking | 25.5 | 24.1 | -1.4(-2.3, -0.5)^c^ | 27.5 | | 24.1 | -3.4(-5.0, -1.9)^b^ | 0.9(0.8, 1.02) | | 0.094 |  |
| Current drinking | 24.1 | 21.5 | -2.6(-3.5, -1.7)^b^ | 23.9 | | 20.4 | -3.5(-5.0, -2.0)^b^ | 0.93(0.84, 1.04) | | 0.194 |  |
| Regular exercise | 33.4 | 45.0 | 11.6(10.5, 12.6)^b^ | 34.4 | | 39.4 | 5.0(3.4, 6.8)^b^ | 1.39(1.28, 1.5) | | <0.001 |  |
| Stress perception | 30.2 | 24.0 | -6.2(-7.1, -5.3)^b^ | 33.9 | | 25.6 | -8.3(-9.9, -6.7)^b^ | 0.93(0.85, 1.02) | | 0.111 |  |
| Overweight or obesity | 39.2 | 40.2 | 1.0(0.0, 2.0) | 34.7 | | 36.6 | 1.9(0.2, 3.7)^c^ | 0.91(0.81, 1.01) | | 0.073 |  |
| Excessive intake of fatty food | 63.5 | 53.0 | -10.5(-11.4, -9.4)^b^ | 61.4 | | 61.1 | -0.3(-2.1, 1.4) | 0.54(0.5, 0.59) | | <0.001 |  |
| Restrictive use of salt | 13.3 | 15.5 | 2.2(1.4, 2.9)^b^ | 13.1 | | 12.4 | -0.7(-1.8, 0.5) | 1.22(1.09, 1.36) | | 0.001 |  |
| **Lifestyle factors (continuous variables)** |  |  |  |  | |  |  |  | |  |  |
| Average number of cigarettes smoked per day | 12.5(7.8) | 13.1(8.2) | 0.6(0.3, 0.9)^b^ | 12.8(7.9) | | 13(8.8) | 0.2(-0.4, 0.7) | -0.51(-0.97, -0.05) | | 0.021 |  |
| Weekly drinking frequency | 2.9(2.3) | 3.0(2.5) | 0.1(0, 0.2)^c^ | 2.9(2.4) | | 2.9(2.3) | 0(-0.1, 0.1) | -0.14(-0.36, 0.09) | | 0.242 |  |
| Weekly exercise duration | 146.2(147.4) | 138.2(166.7) | -8.0(-16.1, 0.1) | 120.5(123) | | 119.4(132.3) | -1.1(-12, 9.8) | 14.07(8.32, 19.79) | | <0.001 |  |

^a^ The multilevel model adjusted age at recruitment, sex, marital status, educational attainment, employment status, workplace-affiliated hospital, history of dyslipidemia, history of diabetes, history of CVD, family history of hypertension, pharmacological treatment; OR, odds ratio; 95%CI, 95% confidence interval; SBP, systolic blood pressure; DBP, diastolic blood pressure; SD: standard deviation; ^b^ *P* < .001. ^c^ *P* < .05 between baseline and 24 months.

| **Table S6. Blood pressure intervention effect on employees with different characteristics** | | | | | | | | |
| --- | --- | --- | --- | --- | --- | --- | --- | --- |
| **Variables** | **Intervention group (n=16,488)** | | | **Control group (n=5,179)** | | | **Intervention Effect^a^** | |
|  | **Baseline, %** | **24 mo, %** | **Change From Baseline, % (95% CI)** | **Baseline, %** | **24 mo, %** | **Change From Baseline, % (95% CI)** | **β(95% CI)** | ***p* value** |
| **SBP mean (SD), mm Hg** |  |  |  |  |  |  |  |  |
| **Education attainment** |  |  |  |  |  |  |  |  |
| Middle school or below | 120.8(11.2) | 120(10.3) | -0.7(-1.1, -0.4)^b^ | 122.1(9.8) | 120.5(9.0) | -1.6(-2.1, -1.0)^b^ | -0.12(-1.10, 0.88) | 0.816 |
| High school | 120.9(11.2) | 119.8(10.2) | -1.1(-1.4, -0.8)^b^ | 121.8(12.0) | 119.6(9.8) | -2.2(-2.7, -1.7)^b^ | -1.38(-2.01, -0.75) | <0.001 |
| College or above | 118.7(12) | 116.8(11.1) | -1.9(-2.2, -1.7)^b^ | 118.6(12.2) | 116.4(11.2) | -2.3(-2.7, -1.9)^b^ | -0.76(-1.28, -0.24) | 0.004 |
| **Employment status** |  |  |  |  |  |  |  |  |
| Manual labor worker | 121.9(11.8) | 119.9(9.8) | -2.1(-2.5, -1.6)^b^ | 121.9(11.8) | 119.9(9.8) | -2.1(-2.5, -1.6)^b^ | -1.04(-1.67, -0.42) | 0.001 |
| Desk job worker | 119.3(11.8) | 117.6(10.9) | -1.6(-1.9, -1.4)^b^ | 118.8(11.7) | 116.5(11.0) | -2.3(-2.7, -1.9)^b^ | -0.91(-1.44, -0.38) | 0.001 |
| Administrative workers | 119.2(12.1) | 117.5(11.2) | -1.8(-2.2, -1.3)^b^ | 120(13.3) | 117.1(11.0) | -2.9(-3.9, -1.9)^b^ | -1.66(-2.72, -0.60) | 0.002 |
| **Workplace-affiliated hospital** |  |  |  |  |  |  |  |  |
| WAH | 119.5(12.0) | 117.8(10.9) | -1.6(-1.8, -1.5)^b^ | 120.4(12.4) | 118.1(10.7) | -2.3(-2.6, -1.9)^b^ | -2.63(-3.53, -1.75) | <0.001 |
| WWH | 122.2(10.7) | 121.0(9.9) | -1.2(-1.5, -0.9)^b^ | 120.6(11.8) | 118.5(10.3) | -2.1(-2.6, -1.6)^b^ | -0.35(-0.77, 0.08) | 0.107 |
| **DBP mean (SD), mm Hg** |  |  |  |  |  |  |  |  |
| **Education attainment** |  |  |  |  |  |  |  |  |
| Middle school or below | 75.3(8.4) | 74.9(8.1) | -0.4(-0.6, -0.1)^b^ | 78.3(6.9) | 77.4(6.8) | -0.9(-1.3, -0.5)^b^ | -0.52(-1.05, 0.00) | 0.051 |
| High school | 76.2(8.5) | 75.6(7.7) | -0.6(-0.8, -0.4)^b^ | 76.9(8.5) | 75.1(7.9) | -1.8(-2.3, -1.4)^b^ | -2.26(-2.79, -1.74) | <0.001 |
| College or above | 74.6(8.7) | 73.9(7.9) | -0.7(-0.9, -0.5)^b^ | 74.2(8.6) | 73.6(7.9) | -0.6(-0.9, -0.3)^b^ | -0.75(-1.16, -0.34) | <0.001 |
| **Employment status** |  |  |  |  |  |  |  |  |
| Manual labor worker | 77.6(8.2) | 76.0(7.6) | -1.6(-1.9, -1.3)^b^ | 77.6(8.2) | 76.0(7.6) | -1.6(-1.9, -1.3)^b^ | -1.85(-2.35, -1.35) | <0.001 |
| Desk job worker | 74.9(8.6) | 74.3(7.8) | -0.7(-0.9, -0.5)^b^ | 74.3(8.0) | 73.8(7.8) | -0.5(-0.9, -0.2)^c^ | -0.40(-0.82, 0.01) | 0.054 |
| Administrative workers | 75.3(9.0) | 74.6(8.0) | -0.6(-1.0, -0.3)^b^ | 76(9.4) | 73.3(8.3) | -2.7(-3.6, -1.9)^b^ | -2.36(-3.25, -1.52) | <0.001 |
| **Workplace-affiliated hospital** |  |  |  |  |  |  |  |  |
| WAH | 75.4(8.9) | 74.7(7.9) | -0.8(-0.9, -0.6) ^b^ | 76.3(8.7) | 74.9(7.8) | -1.4(-1.7, -1.2) ^b^ | -1.93(-2.64, -1.22) | <0.001 |
| WWH | 75.1(8.0) | 74.5(8.0) | -0.6(-0.8, -0.3) ^b^ | 74.8(8.2) | 74.5(7.7) | -0.3(-0.7, 0.1) | -0.88(-1.21, -0.56) | <0.001 |

OR, odds ratio; 95%CI, 95% confidence interval; SBP, systolic blood pressure; DBP, diastolic blood pressure; SD: standard deviation; ^a^ The multilevel model adjusted for age at recruitment, sex, marital status, educational attainment, employment status, workplace-affiliated hospital, history of dyslipidemia, history of diabetes, history of CVD, family history of hypertension, pharmacological treatment; ^b^ *P* < .001. ^c^ *P* < .05 between baseline and 24 months.
